# Photovoltaic properties of halide perovskites for solar cell application with efficiency greater than 18%

**DOI:** 10.1039/d4ra04462b

**Published:** 2024-09-24

**Authors:** Shahid Mehmood, Numan Khan, Zahid Ali, Imad Khan, Sarah Abdullah Alsalhi

**Affiliations:** a Department of Physics, University of Malakand Chakdara Dir 18800 Pakistan Zahidf82@gmail.com shahiduom07@gmail.com; b Collage of Science, Department of Physics, Princess Nourah bint Abdulrahman University Riyadh Saudi Arabia

## Abstract

The opto-electronic properties and solar cell efficiency of halide perovskites A_2_LiInBr_6_ (A = Rb, Cs) are investigated using density functional theory (DFT) through WEIN2k and SCAPS-1D. The electronic characteristic of A_2_LiInBr_6_ (A = Rb, Cs) compounds reveal their direct bandgap semiconductor nature and are active in visible rang. The results indicate that substituting Cs with Rb causes a slight narrowing of the bandgap. According to the optical analysis, these compounds possess dynamic visible-range optical properties that make them ideal for application in opto-electronic devices and solar cells. The A_2_LiInBr_6_ (A = Rb, Cs) absorber layer is employed to simulate the solar cell efficiency of these lead free perovskite-based device. The optimized FTO/WS_2_/A_2_LiInBr_6_ (A = Rb, Cs)/Spiro-MeOTAD/Cu solar cells exhibit the best performance with WS_2_ as the ETL and Spiro-MeOTAD as the HTL having *V*_oc_ value of 2.27 V and 1.85 V, *J*_sc_ value is 11.35 and 11.44 mA cm^−2^, FF is 73.24% and 83.84%, PCE is 18.88% and 17.97%, *R*_s_ is 9.94 and 4.88 Ω cm^2^ and *R*_sh_ is 1.35 and 1.14 Ω cm^2^ respectively. As a result, this research paves the way for future experiments to create entirely inorganic perovskite photovoltaics, free of lead toxicity and exhibit improved photovoltaic ability.

## Abstract 2

The opto-electronic properties and solar cell efficiency of halide perovskites A_2_LiInBr_6_ (A = Rb, Cs) are investigated using density functional theory (DFT) through WEIN2k and SCAPS-1D.

## Graphical abstract

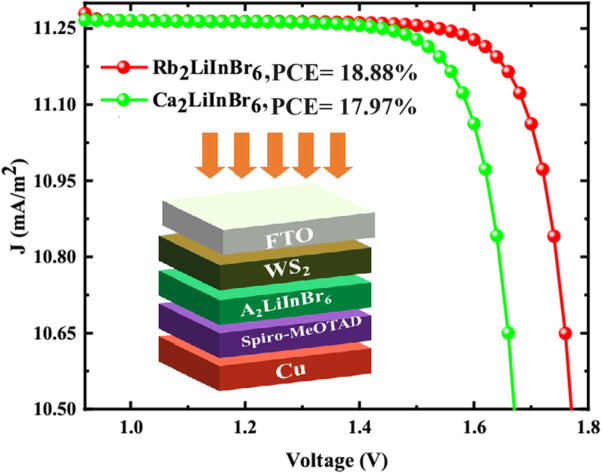

## 1. Introduction

Perovskites, especially double perovskites, have shown great promise in various applications such as solar cells (SCs), light emitting diodes (LEDs), ferroelectrics, photo detectors, electro catalysts, nano-lasers, magneto resistive materials, white light emitters, and spintronics.^1–8^ A lot of the uses of perovskites depend on stability over the long term and inorganic materials are prominent for their robust stability in both air and water, hold significant attraction and promise across various applications.^9^ Numerous applications in optoelectronics, like single junction solar cells are preferably designed with band gaps that align well with the near infrared spectrum. Conversely, most perovskites and double perovskites identified to date show metallic properties or have band gaps within the visible range. Consequently, it is imperative to fabricate new types of perovskites materials, particularly double perovskites with lower band gaps, to facilitate the implementation of numerous practical devices.

Improvements in perovskite solar cells have been dramatic over the last several decades, with efficiency jumping from 3.8% in 2009 to a whopping 22.7% in 2017-all under controlled laboratory conditions.^10–13^ Researcher has shown interest in the latest developments in halide perovskites, especially inorganic-lead halide perovskites like CH_3_NH_3_PbI_3_, because of their amazing optical and photovoltaic properties.^14^ However, issues like lead toxicity and long-term stability concerns hinder the practical use of these materials, hence research into lead-free alternatives is necessary. Exceptional optoelectronic characteristics, low cost, and ease of recycling are required of these substitutes. In addition to competing with existing PV technologies, they also need to fulfill commercial needs including scalability, stability, reliability, and flexibility.^15–17^

In response to these concerns, there has been considerable effort to replace lead-based perovskites with more eco-friendly perovskites based on lead-free halides. The exceptional optoelectronic characteristics of semiconducting halide double perovskites, such as high optical absorption coefficient, elevated carrier mobility, tunable bandgap, minimal electron–hole effective masses, and extensive molar extinction coefficient, have garnered significant interest from materials scientists. Consequently, lead-free alternatives have emerged as a important point in both theoretical and experimental investigations, owing to their stability and potential applications in optoelectronic devices.^18–20^

Due to the exceptional optoelectronic capabilities of the lead-free alternatives, it have shown an effective replacement. First-principles approaches have extensively explored detailed combinations of elements such as Li, K, Na, Cs, In, Ag, Bi, Sb, and halogens within the framework of double perovskites research. These studies line up understanding the diverse uses and potential effectiveness of these compounds.^21–24^ Compounds synthesized by combining of Li, Na K with Ga, In and Tl have recently gained attention because to their advantageous electron–hole carrier mobility characteristics, direct band gap features, and intrinsic stability. Furthermore, significant research accomplishments have focused on first-principles methods to explore lead free perovskites such as Cs_2_InGaX_6_ (X = Cl, Br, or I),^25^ Cs_2_MGaBr_6_ (M = Li, Na),^26^ A_2_KGaI_6_ (A = Cs, Rb),^27^ Rb_2_GaInX_6_ (X = Cl, Br, I),^28^ Rb_2_XGaBr_6_ (X = Na, K)^29^ and Cs_2_AgSb_1−*x*_Ga_*x*_I_6_.^30^ Recently Luo *et al.*^31^ and Sun *et al.*^32^ studied the Li-based double perovskites, Cs_2_LiInX_6_ (X = F, Cl, and Br), Rb_2_LiInBr_6,_ Cs_2_B′In_0.75_Bi_0.25_I_6_ (B′ = Li, Na and K), and reported that these compounds have certain application in optoelectronic devices, additionally their modest electron–hole carrier mass, direct band gap properties thermal and dynamic stability make them excellent candidates for use in optoelectronics and transport dynamics. The investigations highlight the remarkable optical performance of these materials in the visible spectrum, which is influenced by their structural stability and optimum band gap values and possibly applicable for a variety of applications. This study uses DFT calculations to understand how Li-base halide double perovskites, behave when they absorb light. Specifically, the investigation focuses on the A_2_LiInBr_6_ (A = Rb, Cs) compounds, aiming to study their potentials like electrical characteristics, light-harvesting capabilities, optical features and (SC) performance.

## 2. Computational detail

The WIEN2k package,^33^ which utilizing the full potential linear augmented plane wave (FP-LAPW) methods to simulate the optoelectronic properties of A_2_LiInBr_6_ (A = Rb, Cs) within DFT.^34^ To improve results while using minimal computational means, various approaches have been tried to deal with exchange–correlation functionals, these contain Generalized Gradient Approximation (GGA),^35^ Tran and Blaha modified Becke–Johnson potential (TB-mBJ)^36^ and Perovskites mBJ (P-mBJ).^37^ In the FP-LAPW method, a muffin-tin (MT) sphere and interstitial region are usefully designed and employ these constructions to serve the essential function of containing charges within the system and stop charges from escaping. In this arrangement, the configuration of the harmonic expansion is determined within a muffin-tin sphere having *R*_MT_ = 10/*K*_Max,_ near *L*_Max_ = 10. The force convergence criterion is set to 1 mRy Bohr^−1^, ensuring accurate structural optimization, while the expected energy difference between iterations is fine-tuned to 0.1 mRy, promoting stability in the calculation process. To adequately sample the Brillouin zone, a dense 13 × 13 × 13 grid of *K*-points is employed, enabling comprehensive coverage of electronic states. Furthermore, the optical properties of these compounds are rigorously determined using the Kramers–Kronig relation, ensuring accurate characterization of their optoelectronic behavior.

WIEN2k is mostly used for electronic structure calculations but has some limitations when it comes to simulating solar cells. Amorphous or disordered materials, which are common in thin-film solar cells, are challenging to accurately model within the WIEN2k framework. Modeling heterojunctions, which are essential for many high-efficiency solar cells, can be computationally demanding and less straightforward in WIEN2k. SCAP1D on the other hand is more suitable option for solar cell simulations as it is specifically designed to model the electrical behavior of semiconductor devices, including solar cells. It can handle heterojunctions and other complex device structures. SCAP1D can often be coupled with electronic structure codes like WIEN2k to obtain material parameters for more accurate simulations. WIEN2k and SCAP1D are complementary tools. WIEN2k provides fundamental material properties, which SCAP1D can then use to simulate device performance. Combining these tools can result in more accurate and comprehensive solar cell modeling, therefore in the current study the SCAP1D is used to estimate the solar cell parameters for the understudy compounds.

The simulation of the solar cell device, composed of FTO/WS2/A_2_LiInBr_6_ (A = Rb, Cs)/CBTS/Cu, is conducted under carefully controlled parameters. Illumination is provided by a light source featuring an A.M. 1.5 spectrums with an intensity of 1000 mW cm^−2^, replicating standard solar irradiance conditions. The simulated temperature is held constant at 300 K to mimic typical operating environments. Dynamic processes within the device are accurately modeled by setting the frequency of the simulation to 1 × 6^16^ Hz. A scanning voltage range spanning from 0 to 1.50 V is employed to comprehensively assess the device's performance under varying operating conditions. These simulations are executed using SCAPS-1D, a robust software tool renowned for its effectiveness in modeling and optimizing solar cells.^38^ The parameters for electron transport layer (ETL), hole transport layer (HTL) are taken from ref. 39 and 40 and summarized in Table 1 along with absorber layer, the interface flaws between the absorber/HTL and absorber/ETL are described in Table 2 and the parameters used for electrode is summarize in Table 3. There is an assumption that there is no reflection of light on any surfaces or contacts. It is stated that the defect is singular, with a characteristic energy value of 0.1 eV.^41,42^

**Table 1:** Simulation parameters for different layers taking A_2_LiInBr_6_ (A = Rb, Cs) perovskite as absorber layer

Parameters	FTO	WS_2_ (ref. 39)	A_2_LiInBr_6_	Spiro-MeOTAD^40^
Thickness (nm)	500	195	800	200
*E*_g_ (eV)	3.5	1.8	3.13, 3.01	3
*χ* (eV)	4.0	3.95	2.6	2.2
*ε*_r_	9.0	13.6	3.19, 3.32	3.0
*N*_c_ (cm^−3^)	10^19^	10^18^	10^22^	2.2 × 10^18^
*N*_v_ (cm^−3^)	10^19^	10^18^	3.1 × 10^21^	1.8 × 10^19^
*V*_Th,e_ (cm s^−1^)	10^7^	10^7^	10^7^	10^7^
*V*_Th,h_ (cm s^−1^)	10^7^	10^7^	10^7^	10^7^
*μ*_e_ (cm^2^ V s^−1^)	5 × 10^1^	5 × 10^2^	9.74 × 10^2^	2.1 × 10^−3^
*μ*_h_ (cm^2^ V s^−1^)	5 × 10^1^	5 × 10^2^	2.1 × 10^2^	2.16 × 10^−3^
*N*_D_ (cm^−3^)	10^15^	7.25 × 10^18^	0	0
*N*_A_ (cm^−3^)	0	0	10^19^	10^18^
*N*_T_ (cm^−3^)	10^15^	10^15^	10^15^	10^15^

**Table 2:** Simulation parameters interface layer

Parameters	Absorber	ETL/absorber	Absorber/HTL
Type of defect	Neutral	Neutral	Neutral
Cross section for electron (cm^2^)	1 × 10^−15^	1 × 10^−15^	1 × 10^−15^
Cross section for electron (cm^2^)	1 × 10^−15^	1 × 10^−15^	1 × 10^−15^
Energetic distribution	Single	Single	Single
Energy level with respect to *E*_v_ (eV)	0.6	0.65	0.65
Characteristic energy (eV)	0.1	0.1	0.1
Total density (cm^−3^)	10^14^	1 × 10^15^	1 × 10^15^

**Table 3:** Contact parameters used in the simulation

Contacts	Back metal contact properties	Back metal contact properties
Metal work function	5.0	4.56
Surface recombination velocity of electron (cm s^−1^)	1 × 10^7^	1 × 10^7^
Surface recombination velocity of electron (cm s^−1^)	1 × 10^5^	1 × 10^5^

## 3. Results and discussion

### 3.1 Electronic properties

Many physical characteristics of compounds are associated to their electronic band structure. As per the literature, each compound possesses a distinct set of electrical characteristics owing to the uniqueness of its band structure.

**Fig. 1:**
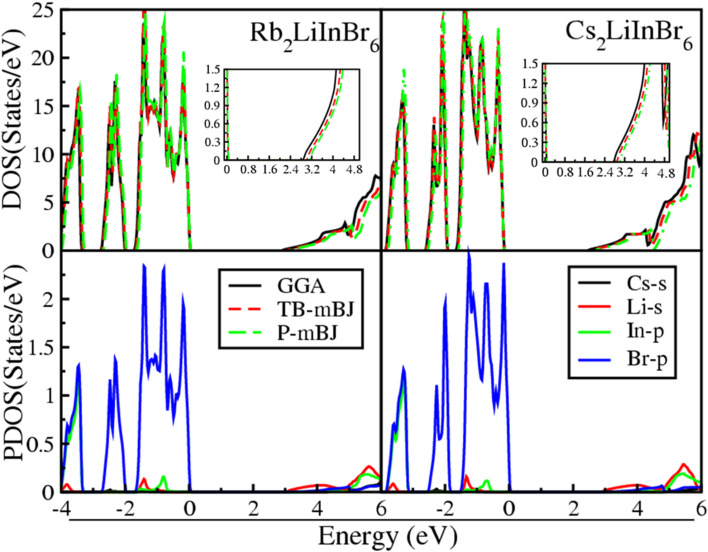
Total DOSs and partial DOSs of the A_2_LiInBr_6_ (A = Rb, Cs) lead free halide perovskites.

The electrical properties of materials are related to their density of states (DOS), representing the distribution of available electronic states at various energy levels. Fig. 1 shows the total density of states (TDOS) obtained through different computational methods, including GGA, TB-mBJ, and P-mBJ. Notably, the Fermi level (*E*_F_) is standardized at 0 eV to accurately assess the TDOS for these compounds, offering a comprehensive understanding of their electronic structures and behavior. The TDOS reveals that the A_2_LiInBr_6_ (A = Rb, Cs) compounds are semiconductors; the valence band (VB) and conduction (CB) encompass the energy gap and *E*_F_ does not cross by densities.

The bandgap values for these compounds were calculated in the current study using several potentials. The band gap value obtained by GGA, TB-mBJ and P-mBJ are 2.88, 3.02 and 3.13 eV for Rb_2_LiInBr_6_ and are 2.76, 2.89 and 3.01 eV for Cs_2_LiInBr_6_ respectively shown in Table 4. Other groups previously calculated the band gap of isotropic double perovskites compounds *e.g.* Cs_2_AlBiCl_6_, Cs_2_LiAlCl_6_, Cs_2_LiInBr_6,_Rb_2_AgGaCl_6_ and Cs_2_AgSbCl_6_ are 3.23 eV, 3.22 eV, 3.75 eV, 2.53 eV and 2.35 eV respectively.^26,31,43^ Cs and Rb are close enough that substituting one for the other won't significantly alter their structure or functionality. The features of these lead-free double perovskites in the current study are similar to those used as solar cell and are less hazardous than lead perovskites. The present study's comparative analysis of estimated band gaps shows good agreement with the band gap of available literature. This agreement confirms the accuracy of mBJ approach's in band gap estimation and due to its usefulness it is used throughout the calculation.

**Table 4:** Calculated band gap of the A_2_LiInBr_6_ (A = Rb, Cs) structured compounds by different approximations

Parameters	GGA	TB-mBJ	P-mBJ	Other
Rb_2_LiInBr_6_	2.88	3.02	3.13	2.6^[mBJ]^^42^
Cs_2_LiInBr_6_	2.76	2.89	3.01	2.2^[mBJ]^, 3.75^[HSE06]^^31,42^

According to the literature, mBJ method is proving to be quite efficient and accurate in determining band gaps for semiconductors. By proposing results comparable to more expensive methods like hybrid functional or GW, while being cost-effective like LDA/GGA functionals, it presents a valuable option for researchers. The improved exchange potential, treatment of localization, and accurate description of dielectric properties. Additionally its ability to provide results closer to experimental values than other theoretical approaches is a significant advantage and seem to be a promising tool for treating semiconductor.^36,37^

The graphical interpretation of Total Density of States (TDOS) for both compounds shows that switching from Rb to cation Cs, the valence states moves towards higher energy is less compared to the movement of conduction states towards lower energy. These results confirm that, the band gap becoming narrower and more precisely tuned. This effect is attributed to the increase in Cs cations ionic radii. Therefore, the ionic radius of mentioned halide ions influences hybridization, that further leading to suppression in the simulated band gap. This suppression in the band gap presents a valuable advantage for practical device fabrication, as it occurs across a range of energy levels, providing versatility in optimizing device performance for various applications.^44^

The Partial Density of States (PDOS) calculations for the lead-free compounds A_2_LiInBr_6_ (A = Rb, Cs) is depicted in Fig. 1, which offers valuable insight into the semiconducting properties of these materials. In Fig. 1 the s state of Cs and Li and p state of In and Br displays clearly. The contribution of both Cs and Li ‘s’ states are negligible but most of the contribution is due to the p state of In and Br. The graph shows that in VB the p state of In and Br contribute mostly and are from −4 to zero eV while the contribution of the Li s state to the CB extends from the band gap to 6 eV correspondingly. The band gap occurs between the Br-p state and Li-s state in both compounds.

**Fig. 2:**
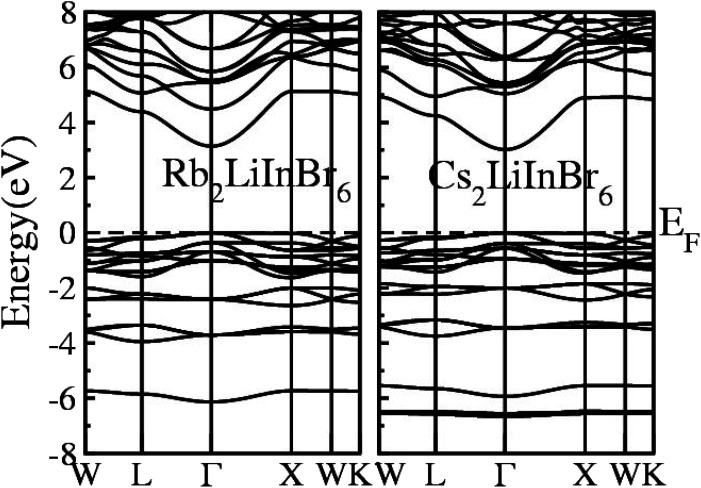
Electronic band structure of the A_2_LiInBr_6_ (A = Rb, Cs) lead free halide perovskites.

The calculated Electronic Band Structure (EBS) plotted across the irreducible Brillouin zone in *K*-space, specifically along high symmetry directions, for the A_2_LiInBr_6_ (A = Rb, Cs) compounds, that provides a detailed representation of the electronic properties of these materials presented in Fig. 2. The EBS analysis for these compounds confirms their semiconducting nature. Specifically the gamma symmetry point, where the VB maxima and CB minima meet signifies their behavior as direct band gap semiconductors. The calculated band gaps for A_2_LiInBr_6_ (A = Rb, Cs) compounds are 3.13 eV and 3.01 eV respectively by utilizing P-mBJ potential given is presented in Table 4. According to findings from another research group, the reported band gaps for these compounds are 2.6 eV and 2.2 eV, respectively, employing the mBJ potential.^44^ In Fig. 2, it's evident that the bandgap of the investigated compounds decreases as we switch from Rb to Cs. This trend is consistent with the substitution of cations with higher electronegativity for those with lower electronegativity. This phenomenon mirrors observations in other compounds, such as A_3_In_2_As_4_ and A_5_In_2_As_6_ (A = Sr and Eu), where the bandgap also decreases.^45^

### 3.2 Optical properties

**Fig. 3:**
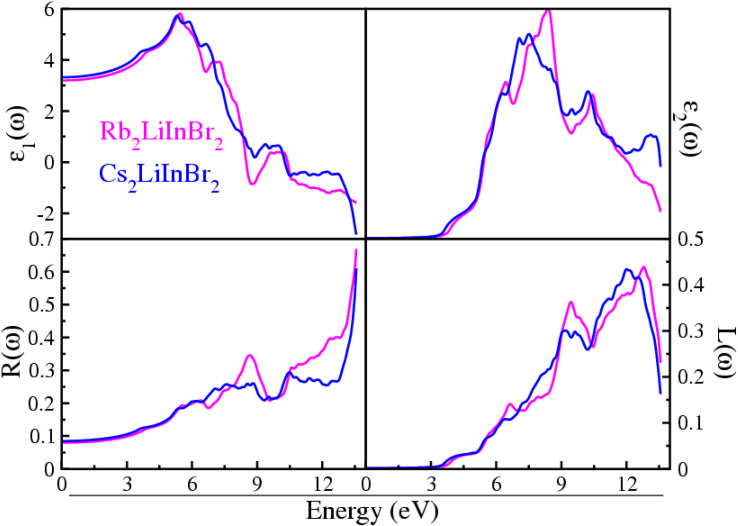
Dielectric function, reflectivity and loss function of the A_2_LiInBr_6_ (A = Rb, Cs) lead free halide perovskites.

**Fig. 4:**
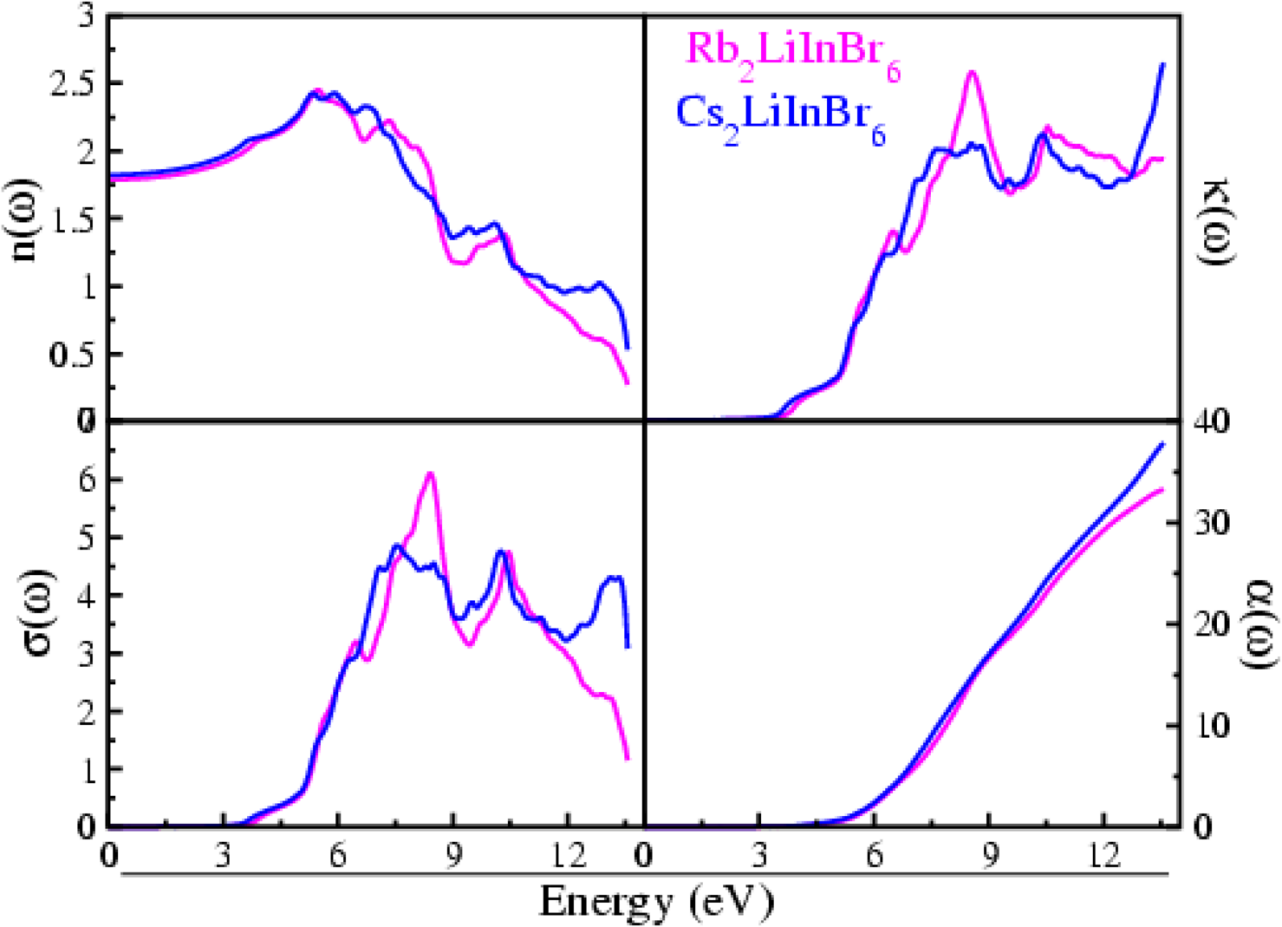
Refractive index, oscillator strength, excitation coefficient and optical conductivity of the A_2_LiInBr_6_ (A = Rb, Cs) lead free halide perovskites.

Understanding the precise nature of interaction of light waves between these materials is of the utmost importance for opto-electronics applications. Here the optical properties are evaluated throughout an energy range of 0 to 14 eV has been carried out on these A_2_LiInBr_6_ (A = Rb, Cs) compounds. The optical factors computed in the current study are visualized in Fig. 3 and 4. These figures help us understand how light interacts with the materials we're studying, giving us a clear picture of their properties and behavior. The computed optical factors incorporate both the real part *ε*_1_(*ω*) and imaginary part *ε*_2_(*ω*) of the dielectric function, reflectivity *R*(*ω*), energy loss function *L*(*ω*), extinction coefficient *k*(*ω*), oscillator strength *α*(*ω*), refractive index *n*(*ω*), and optical conductivity *σ*(*ω*). These factors offer a thorough understanding of how light interacts with and characterizes the materials under investigation, providing valuable insights into their optical properties and behavior.

The physical properties of the compound are described by the dielectric real part *ε*_1_(*ω*) are shown in In Fig. 3. The static dielectric constant, often denoted as *ε*_1_(0), represents the value of the dielectric function at zero electron volts (0 eV). This parameter provides crucial insight into the material's response to electric fields and its overall electronic structure. The static dielectric function *ε*_1_(0) provides insight into the material's polarization behavior, especially at low frequencies, reflecting the energy levels involved in external interactions. The calculated *ε*_1_(0) results for Rb_2_LiInBr_6_ and Cs_2_LiInBr_6_ compounds which are shown in Fig. 3, are 3.19 eV and 3.32 eV respectively. In A_2_LiInBr_6_, when A is switched from cation Rb to cation Cs, the value of *ε*_1_(0) increases while the band gap declines. This trend aligns strongly with Penn's model, which states that *ε*_1_(0) = 1 + [*hω*_p_/*E*_g_].^44^ The simulated values of *ε*_1_ (0) and band gape (*E*_g_) exhibit an inverse relationship, that confirming Penn's model. For A_2_LiInBr_6_ (A = Rb, Cs) compounds peaks arises at 5.45 and 5.29 eV, respectively, are observed in the spectra following at zero frequency. The spectra progressively flatten down up to become zero after this peak and eventually approach zero behaving like metal at those energies and no longer dielectric.

Fig. 3 illustrates the *ε*_2_(*ω*) values, indicating that there is no spectra present up to the optical gaps also listed in Table 3, which are 3.55 eV and 3.36 eV. The presence of multiple peaks in the imaginary dielectric function suggests that electronic excitations are influenced not just by energy levels but also by the calculated electronic properties of the material. These findings imply a deeper comprehension of the complicated relationship between electronic behavior and its manifestation in optical properties.

The calculated optical gap contracts when transitioning from Rb to Cs and are visible-light active, therefore suitable for solar cell application. The compounds A_2_LiInBr_6_ (A = Rb, Cs) in In Fig. 3 just after the threshold value, the curve begins to increase sharply, reaching peaks at 8.30 and 7.49 eV correspondingly, this enhanced understanding arises from significant interband transitions within the material.

The calculated reflectivity *R*(*ω*) for A_2_LiInBr_6_ (A = Rb, Cs) compounds is shown in Fig. 3 which is simply the fraction of reflected energy to incidental energy, serves to briefly reveal how a surface reacts to the incoming energy. As the 0 eV reflectance *R*(0) for A_2_LiInBr_6_ (A = Rb, Cs) compounds ranges from 7 to 8% respectively. The *R*(0) for Rb_2_LiInBr_6_ exhibits an extreme reflectivity of 67% at 13.56 eV, while for Cs_2_LiInBr_6_; the high reflectivity is 61% sharply at 13.6 eV. In the current study, as the energy approaches the point where the dielectric function *ε*_1_(*ω*) tends towards zero, there is a corresponding increase in reflectivity. This observation suggests that as the material's electronic properties align with certain energy levels, it becomes more reflective to incident light. The high reflectivity observed in the ultraviolet (UV) spectrum suggests that these compounds could serve effectively as a shield against high-frequency radiation.

The energy loss function, *L*(*ω*), which provides understanding into the energy dissipation of electrons pass through the material.^46,47^ Due to plasma frequency resonance, the studied compounds curves attain a maximum peak. Scattering does not occur where energy of photons is higher than the bandgap value, as seen in Fig. 3. Also Fig. 3 shows that the loss energy of the A_2_LiInBr_6_ (A = Rb, Cs) compounds extends from 9.10 to 13.07 eV, respectively, because of discrete energy bands.^48^

The *n*(*ω*) represent refractive index for the compounds understudy, presented in Fig. 4. For Rb_2_LiInBr_6_, the *n*(*ω*) value at zero frequency, that is *n*(0) is 1.78 although for Cs_2_LiInBr_6_, it is 1.82. After a certain point, the *n*(*ω*) real component starts to rise up to reaches its peak value, indicating the highest energy value which is 5.48 eV for Rb_2_LiInBr_6_ and 6.73 eV for Cs_2_LiInBr_6_.These compounds show significant refractive index *n*(*ω*) values, are a result of the limited passage of photons through the materials, indicating their substantial optical density.^4^

The material's ability to allow the passage of electrons when subjected to an electromagnetic field is quantified by its optical conductivity, denoted as *σ*(*ω*),which is presented in Fig. 4 for A_2_LiInBr_6_ (A = Rb, Cs) compounds, The *σ*(*ω*) initiates at the bandgap and progressively increases beyond it. A_2_LiInBr_6_ (A = Rb, Cs) compounds achieve their *σ*(*ω*) with respective values of 6.10 and 4.86 Ω^−1^ cm^−1^, as shown in Fig. 4.

The extinction coefficient is a measure of the interaction between a material and an electromagnetic wave, represented as *k*(*ω*).^49–53^ It's a practical conclusion that a material with a low *k*(*ω*) would exhibit excellent transmittance and minimal absorption.

The *k*(*ω*) values are predominantly zero up to the band gap, indicating minimal absorption and strong transmittance as demonstrated in Fig. 4. Maximum value for Rb_2_LiInBr_6_ is 1.72 at 8.55 eV and for Cs_2_LiInBr_6_ is 1.76 at 13.56 eV; these findings indicate that at these energy levels, both compounds exhibit high wave absorption and low wave transmission and is closely aligns with *ε*_2_(*ω*).

The oscillator strength, represented as *α*(*ω*), plays a crucial role in determining the number of electrons within the material that engage in an optical transition. As energy increases, the oscillator strength function *α*(*ω*), as shown in Fig. 4 for these compounds, also rises. The absence of occupied bands at lower energies can be attributed to the zero-sum rule. This principle indicates that the total number of electrons in the system remains constant; leading to the empty states in the unoccupied bands at lower energies. As here the bandgap narrows, there's a corresponding increase in the oscillator strength *α*(*ω*). As energy levels increase, there is a rapid escalation in the electron count within a system, starting from a zero baseline at lower energy states. The oscillator strength *α*(*ω*) analysis suggests that approximately 33–38 electrons participate in the optical transitions for the A_2_LiInBr_6_ (A = Rb, Cs) compounds, as observed from Fig. 4.

In summary, the distinguishing optical and electronic properties exhibited by these compounds underscore their promising suitability for utilization in solar cell technology.

### 3.3 Modelling and simulation of solar cell device

The optical properties exhibited by the investigated compounds prompt a deeper exploration of their potential as solar cell materials, particularly through an assessment of solar cell efficiency and current density (*J*) *versus* voltage (*V*) performances using the Solar Cell Capacitance Simulator (SCAPS – 1D).^38^ To assess the performance of solar cells constructed from several different double perovskites structures, we examine the impact of absorber layers and varying thicknesses of ETL. In this investigation, contour plot mapping is employed to assess key performance parameters of solar cells. The selection of an appropriate absorber material, HTL and ETL is crucial for attaining highest performance solar cells. By investigative contour plots representing efficiency, fill factor, open-circuit voltage, and short-circuit current density, researchers can identify optimal combinations of absorber, HTL and ETL materials that maximize solar cell performance. Additionally, selecting the appropriate thickness for the absorber, HTL and ETL is crucial for effectively capturing light and collecting holes generated within the absorber layer.

In the proposed simulated structures, Ws_2_ and TiO_2_–SnO_2_ as taken as ETL and NiO, CuSCN, CuI, MoO_3_, WSe_2_, Spiro-MeOTAD and Cu_2_BaSnS_4_ denoted by (CBTS) as HTL, in addition Cu is used as a metallic back contact were tasted and the parameters including the short circuit current density (*J*_SC_), open circuit voltage (*V*_OC_), fill factor (FF), and PCE and the results are shown in Table 5.

**Table 5:** The comparison of PV parameters of A_2_LiInBr_6_ (A = Rb, Cs) with variation in HTL and ETL

Devise structure	HTL	ETL	*V*_OC_ (V)	*J*_C_ (mA cm^−1^)	FF (%)	PCE (%)
FTO/NiO/absorber/WS_2_/Cu	NiO	WS_2_	1.31 (0.98)	16.71 (17.59)	78.26 (72.42)	17.25, (12.49)
FTO/CuSCN/absorber/WS_2_/Cu	CuSCN	WS_2_	1.31 (0.98)	16.64 (17.59)	78.22 (72.42)	17.17 (12.50)
FTO/CuI/absorber/WS_2_/Cu	CuI	WS_2_	1.31 (0.98)	16.64(17.58)	77.97 (72.43)	17.11 (12.49)
FTO/MoO_3_/absorber/WS_2_/Cu	MoO_3_	WS_2_	1.31 (0.98)	16.64 (17.59)	78.26 (72.43)	17.29 (12.50)
FTO/WSe_2_/absorber/WS_2_/Cu	WSe_2_	WS_2_	1.31 (0.98)	16.72 (17.58)	78.27 (72.43)	17.27 (12.50)
FTO/Spiro-MeOTAD/absorber/WS_2_/Cu	Spiro-MeOTAD	WS_2_	2.27 (1.85)	11.35 (11.44)	73.24 (83.84)	18.88 (17.97)
FTO/CBTS/absorber/WS_2_/Cu	CBTS	WS_2_	1.61 (1.53)	16.41 (16.42)	70.82 (71.44)	18.77 (17.94)
FTO/CBTS/absorber/TiO_2_–SnO_2_/Cu	CBTS	TiO_2_–SnO_2_	1.23 (1.12)	6.60 (12.89)	84.39 (81.68)	6.88 (11.81)

**Fig. 5:**
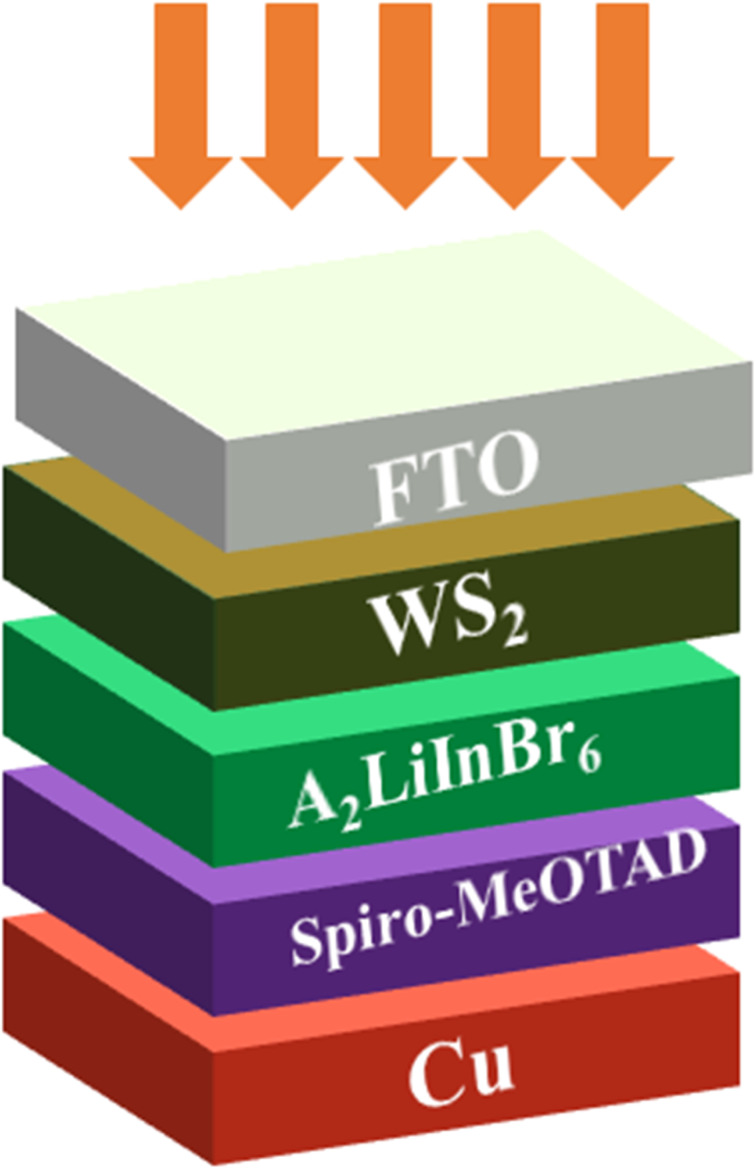
Schematic diagram of the solar cell device having A_2_LiInBr_6_ (A = Rb, Cs) lead free halide perovskites as a active layer.

**Fig. 6:**
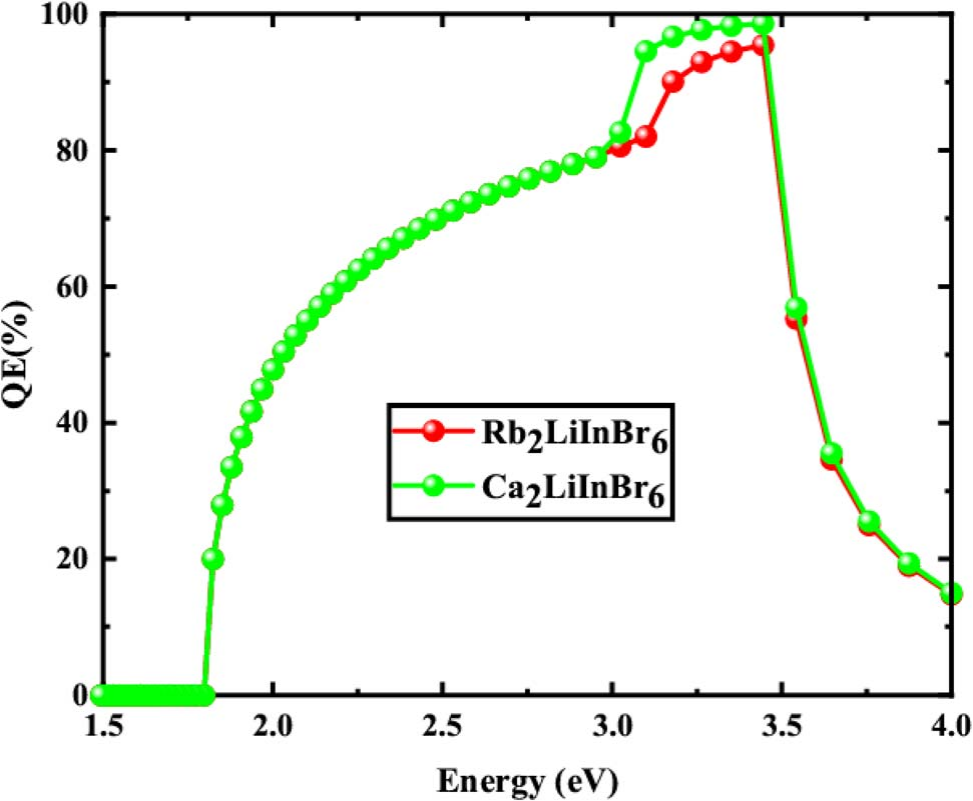
Quantum efficiency against photon wavelength of the solar cell device.

**Fig. 7:**
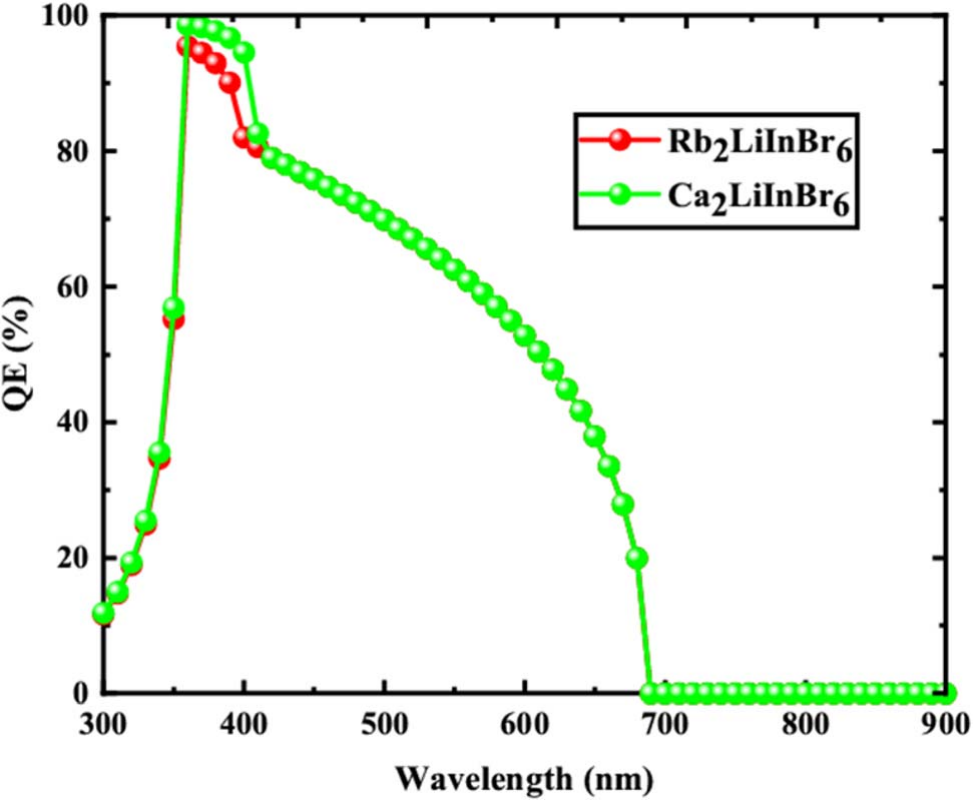
Quantum efficiency against photon energy of the solar cell device.

From the Table 5 the finest solar cell performance is attained for FTO/WS_2_/A_2_LiInBr_6_ (A = Rb, Cs)/Spiro-MeOTAD/Cu and their proposed structure as shown in Fig. 5. The quantum efficiency (QE) of a solar cell measures how effectively it converts incident photons into electron–hole pairs, expressed as the ratio of captured pairs to incident photons. These performance metrics are often characterized as functions of photon energy in electron volts (eV) or wavelength in nanometers (nm).^54^ The wavelength range from 300 to 900 nm and the photon energy range from 1.3 to 4.13 eV are utilized to measure the quantum efficiency (QE), as illustrated in Fig. 6 and 7, respectively. When using A_2_LiInBr_6_ compounds (A = Rb, Cs) as an absorber which is the key layer, the simulated device's quantum efficiency (QE) ranges from 11.67% to 11.98% at 4.1 eV (300 nm) and reaches 96 and 99% at 3.44–3.35 eV (360–370 nm) respectively and decreased to 19.96 and 19.98% at 1.82 eV (680 nm). Through careful analysis of the visible spectrum, the device is able to effectively convert photon energy into electrical energy. Nevertheless, the quantum efficiency (QE) reached to zero as the wavelengths become longer and the energy levels decrease. The HTL plays a crucial role in shaping the photovoltaic characteristics and hinders the movement of electrons, minimizing quenching losses by preventing effectively electron–hole (e–h) recombination and facilitating the drift of generated holes from the perovskite absorber layer to the back metallic contact, thereby enhancing power conversion efficiency (PCE).

**Fig. 8:**
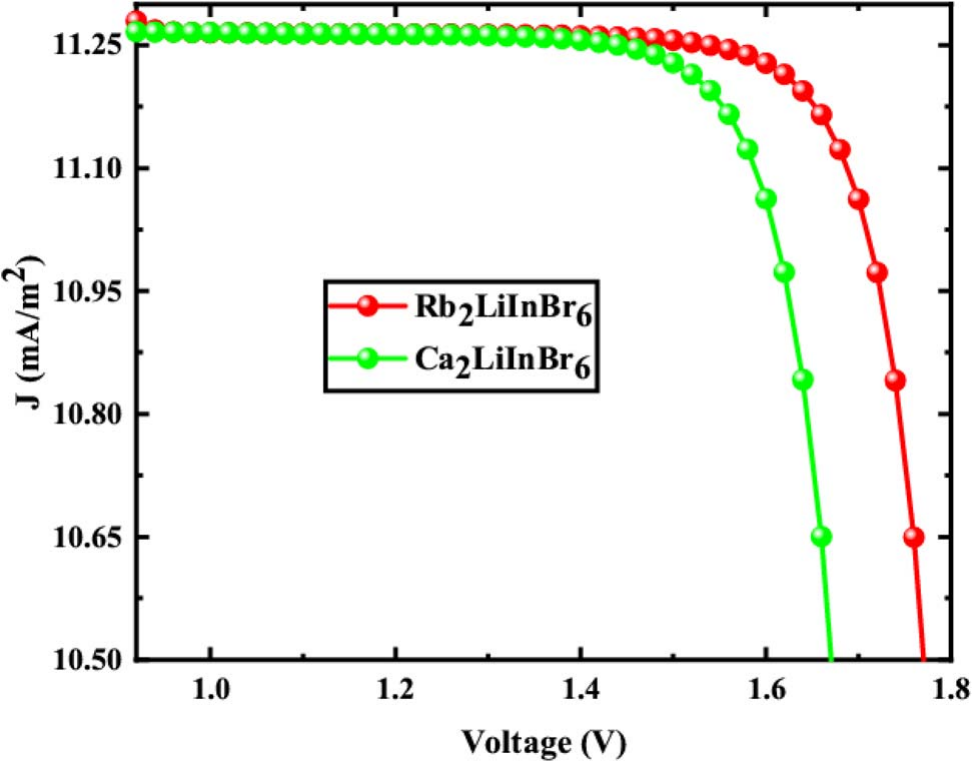
*J versus V* characteristic of the solar cell device.

The solar cell parameters like *V*_OC_, *J*_SC_, FF, PCE, series resistance (*R*_s_) and shunt resistance (*R*_sh_) are obtained from the *J*–*V* shown in Fig. 8 for FTO/WS_2_/A_2_LiInBr_6_ (A = Rb, Cs)/Spiro-MeOTAD/Cu device, and the data is provided in Table 6, which is used to evaluate the photovoltaic (PV) characteristics.

**Table 6:** *J*–*V* characteristic parameters for A_2_LiInBr_6_ (A = Rb, Cs)

Parameters	Rb_2_LiInBr_6_	Cs_2_LiInBr_6_
*V*_OC_ (V)	2.27	1.85
*J*_C_ (mA cm^−1^)	11.35	11.44
FF (%)	73.24	83.84
PCE (%)	18.88	17.97
*R*_s_	9.64	4.88
*R*_sh_	1.35	1.14

From the Table 6 the *V*_OC_ is 2.27 V and 1.85 V, *J*_SC_ is 11.35 and 11.44 mA cm^−2^, FF is 73.24% and 83.84%, PCE is 18.88% and 17.97%, *R*_s_ is 9.94 and 4.88 Ω cm^2^ and *R*_sh_ is 1.35 and 1.14 Ω cm^2^ correspondingly. When the bandgap of the active layer increases, it absorbs fewer photons, which in turn lowers the short-circuit current density (*J*_SC_). However, this also leads to an increase in the open-circuit voltage (*V*_OC_) because charge carriers segregate more easily within the active layer. These alterations have a significant impact on the overall power conversion efficiency (PCE) of the solar cell. As a consequence, the PCE of Rb_2_LiInBr_6_ is higher than that of the Cs_2_LiInBr_6_ base active layer.

**Fig. 9:**
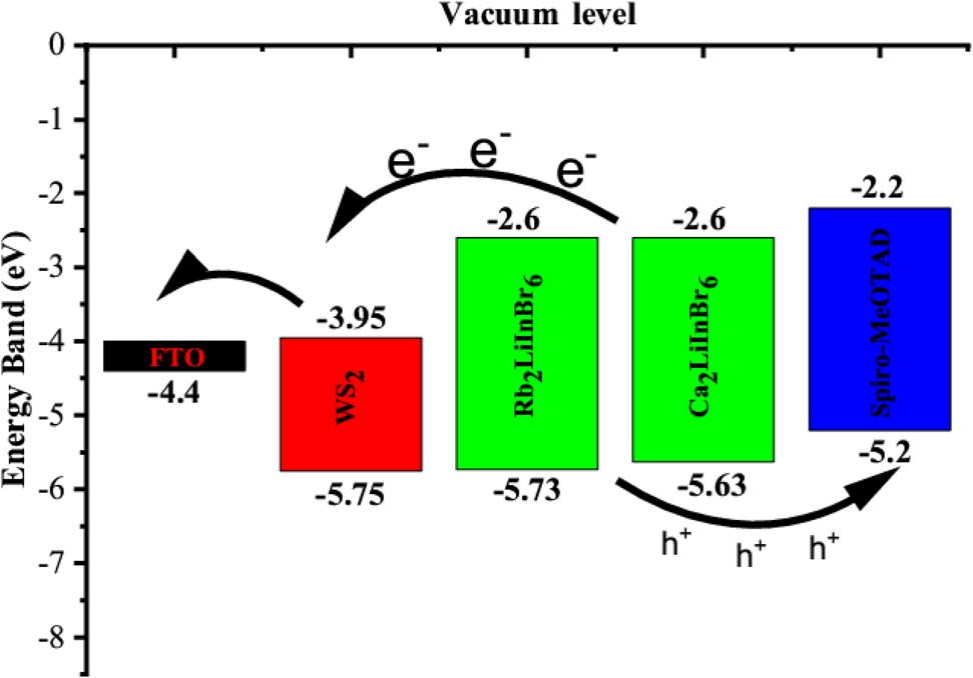
Schematic diagram of charge transfer mechanism in of the solar cell device.

The valence band maxima (VBM) and conduction band minima (CBM) of the absorber, HTL and ETL layers are displayed in Fig. 9. The results demonstrate that the energy disparity between the absorber layer and the CBM of WS_2_ is less than that between the absorber layer and the VBM of WS_2_. Consequently, it promotes electron conduction from the absorber's conduction band to the FTO *via* the WS_2_ layer while inhibiting hole conduction from the absorber's VBM to the WS_2_ layer's VBM. In a similar vein, the energy disparity between the absorber layer's CBM and Spiro-oMeTAD is greater than the VBM's disparity with Spiro-MeOTAD. Due to these distinctions, holes can easily pass from the absorber's VBM to the Spiro-MeOTAD's VBM, but electrons can't pass from the absorber's CBM to the Spiro-MeOTAD's CBM.

Comparative study of the SCAPS-1D outcomes with reported work by using different double perovskites as an absorber layer is presented in Table 7. This comparison allows for an assessment of our work in relation to existing research. The table indicates that all the mentioned absorber layers did not achieve the awaited efficiency levels in previous studies. However, the current optimized device demonstrates significantly higher efficiency compared to previous works, highlighting the effectiveness of the current study. By integrating the optimized values obtained from this study with other reported results, it is observed that a substantial increase in the Power Conversion Efficiency (PCE) occur simulated in the current study. The previous reported PCE in Table 5 are 16.35%,^55^ 1.965%,^56^ 12%,^43^ 10.30% (ref. 57) and 2.39% (ref. 58) while the PCE of the reported lead free absorber layer is 18.88 and 17. 97% respectively. This noteworthy improvement underscores the significance of this study in which environmentally friendly, Pb-free Perovskites Solar Cell (PSC) is designed, which incorporates inorganic charge transport layers.

**Table 7:** The comparison of PV parameters of A_2_LiInBr_6_ (A = Rb, Cs) and similar absorbers-based solar cells

Absorber	PCE (%)	Ref.
Cs_2_GeSnCl_6_	16.35	53
Cs_2_AlBiCl_6_	1.965	54
Rb_2_AgGaCl_6_	12	41
Cs_2_CuBiCl_6_	10.30	55
Cu_2_AgBiI_6_	2.39	56
Rb_2_LiInBr_6_	18.88	This work
Cs_2_LiInBr_6_	17.97	This work

## 4. Conclusions

The optoelectronic and photovoltaic characteristics of A_2_LiInBr_6_ (A = Rb, Cs) compounds are studied utilizing the FP-LAPW scheme in a DFT using GGA, TB-mBJ and P-mBJ potentials. Their band structure, total and partial densities of state calculations reveals that the A_2_LiInBr_6_ (A = Rb, Cs) are direct band gap semiconductors and the band gap values for A_2_LiInBr_6_ (A = Rb, Cs) drop from 3.13 to 3.01 eV when the cation is changed from Rb to Cs. The findings indicate that the examined materials exhibit optical dynamics within the visible range of the electromagnetic spectrum. This observation underscores their potential suitability for use as Solar Cell devices. The Solar Cell performance of device based on these perovskites is simulated by utilizing WS_2_ as the electron transport layer (ETL), Spiro-MeOTAD as the hole transport layer (HTL), and A_2_LiInBr_6_ (A = Rb, Cs) as absorber layer using SCAPS-1D. In the optimized FTO/WS_2_/A_2_LiInBr_6_ (A = Rb, Cs)/Spiro-MeOTAD/Cu solar cell structure, the achieved values are as follows: as *V*_OC_ of 2.27 V and 1.85 V, *J*_SC_ value is 11.35 and 11.44 mA cm^−2^, FF is 73.24% and 83.84%, PCE is 18.88% and 17.97%, *R*_s_ is 9.94 and 4.88 Ω cm^2^ and *R*_sh_ is 1.35 and 1.14 Ω cm^2^ respectively. For these reasons, our research covers the way for future experiments to develop inorganic lead-halide perovskites with enhanced photovoltaic performance.

## Data availability

The data used in the current study are available from the corresponding author on reasonable request.

## Conflicts of interest

The authors declare that they have no competing interest.

## Author contributions

Shahid Mehmood: investigation, methodology, writing – reviewing and editing, Numan khan: investigation, data curation, methodology, writing – original draft preparation, Zahid Ali: project administration, supervision, conceptualization, Imad Khan: visualization, formal analysis, reviewing and editing, Sarah Abdullah Alsalhi: funding acquisition, resources, software.
